# Characterization of the Second-Generation Covalent Fragment Library (CovLib Gen2): Thiol Reactivity Profiling and p53-Y220C Rescue

**DOI:** 10.2147/DDDT.S598622

**Published:** 2026-05-18

**Authors:** Martin Schwer, Sven R Aldea, Marc U Engelhardt, Jason Stahlecker, Janosch Rheinganz, Aaron Langkamp, Frank M Boeckler

**Affiliations:** 1 Department of Pharmacy and Biochemistry, Laboratory for Molecular Design & Pharmaceutical Biophysics, Eberhard Karls Universität Tübingen, Institute of Pharmaceutical Sciences, Tübingen, 72076, Germany; 2 Interfaculty Institute for Biomedical Informatics (IBMI), Eberhard Karls Universität Tübingen, Tübingen, 72076, Germany

**Keywords:** covalent fragment-based drug discovery, differential scanning fluorimetry, 5, 5’-dithiobis-(2-nitrobenzoic acid), glutathione, tumor suppressor p53, warheads

## Abstract

**Purpose:**

Covalent Fragment-Based Drug Discovery (FBDD) has emerged as a powerful strategy for unlocking challenging pharmacological targets and engaging shallow or “cryptic” binding pockets. In this study, we present the design and characterization of the Second Generation Covalent Fragment Library (CovLib Gen2), an expanded collection of 81 structurally diverse electrophiles tailored for Covalent Fragment-Based Drug Discovery (FBDD) using an electrophile-first approach. The library spans five distinct warhead classes, including epoxides, vinyl sulfones, acrylamides, α-cyanoacrylamides, and a core set of SɴAr-reactive heteroarenes.

**Methods:**

We comprehensively profiled the library for physicochemical properties and intrinsic thiol reactivity using high-throughput 5,5’-dithiobis-(2-nitrobenzoic acid) (DTNB) and high-performance liquid chromatography (HPLC)-based glutathione (GSH) reactivity assays. To demonstrate the library’s utility, we performed differential scanning fluorimetry (DSF) screening against the oncogenic, thermally unstable p53-Y220C mutant and subsequent specificity testing with two control mutants.

**Results:**

The library exhibited a broad dynamic range of reactivities with a clear correlation between the assay methods. Additionally, we identified 12 fragments with desirable mild reactivity profiles (t_1/2_GSH = 1–10 h). The DSF screen yielded 15 hits, primarily SɴAr-reactive heteroarenes and vinyl sulfones. Notably, the fragment SN054 emerged as the most potent stabilizer, inducing a maximal thermal shift of 4.5 °C. Specificity was confirmed using a cysteine-light variant (T-p53C-Y220C-CL), where SN054 retained significant stabilizing activity.

**Conclusion:**

Our findings validate CovLib Gen2 as a versatile tool for ligand discovery, including electrophilic fragments covering a broad range of reactivity, and provide tractable starting points for the pharmacological rescue of p53-Y220C.

## Graphical Abstract

**Figure:**
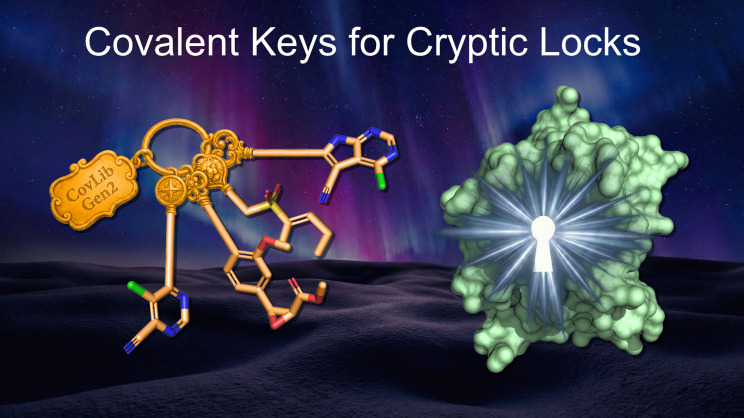

## Introduction

The landscape of drug discovery has been significantly impacted by the resurgence of targeted covalent inhibitors (TCIs). While historical concerns regarding off-target toxicity persisted, modern TCI design has demonstrated that covalent binding offers distinct advantages, including high potency, prolonged duration of action, and the ability to target shallow binding pockets.^1–10^ Their success is underlined by the FDA approval of several covalent-acting drugs, primarily kinase inhibitors, as well as antiviral drugs like Nirmatrelvir, used for the treatment of COVID-19.^8^,^11–16^ Typically, these inhibitors function via a two-step mechanism: initial reversible association followed by the formation of a covalent bond through the reaction of a covalent reactive group (CRG, often referred to as “warhead”) with a nucleophilic residue, most commonly cysteine.^17–19^ The standard TCI design strategy relies on a structure-based approach, where a warhead is attached to a known non-covalently binding ligand to target a specific nucleophilic amino acid residue.^9^,^19–22^ Moreover, covalent fragment-based drug discovery (FBDD) has emerged as a powerful complementary strategy. Often termed the “electrophile-first” approach, covalent FBDD screens small, reactive fragments to identify novel binding sites—including allosteric or transient protein binding sites—without relying on a pre-existing non-covalent ligand binding next to a targetable amino acid residue.^23–34^

Previously, our group developed a halogen-enriched fragment library (HEFLib) intended to investigate the application of halogen bonding in FBDD.^35–39^ During characterization, we observed that one specific fragment (compound 4482) stabilized the tumor suppressor p53 not through non-covalent interactions, but by covalently modifying surface cysteines via nucleophilic aromatic substitution (SɴAr).^38^ This fragment is identical to the compound PK11000, which was previously shown by Bauer et al to covalently bind and stabilize p53-Y220C.^40^ In principle, halogen bonding is driven by the interaction between a Lewis base and the electron-deficient σ-hole of a halogen atom.^41^,^42^ While the strength of this interaction (V_max_) can be enhanced by adding electron-withdrawing groups to the aromatic scaffold, increasing the electrophilicity carries the risk of triggering unintended nucleophilic aromatic substitution (SɴAr) at the arene core, leading to the observed unintended protein arylation.^43–45^ This reveals not only the necessity for thiol-reactivity profiling of electron-deficient aromatic fragments, but highlights the potential of S_N_Ar-reacting heterocycles as a warhead class additional to the frequently used Michael-type acceptors. Building on these observations, in addition to the previously mentioned advantages of TCIs and covalent FBDD, we aimed to design a diverse Covalent Fragment Library (CovLib).^46^ Effective covalent library design merges classical FBDD parameters^47^ (“rule of three”) with warhead characteristics like reactivity, stability, and reaction geometry. To ensure maximum diversity, the library should feature diverse warhead classes spanning a wide dynamic range of reactivities.^23^,^25^,^48^,^49^ Due to the pandemic and other restrictions, we started our CovLib with a first subset comprising 20 fragments across four warhead classes: α-cyanoacrylates, epoxides, vinyl sulfones, and SɴAr-reactive heteroarenes. The physicochemical profiling of this subset, including computational and experimental solubility assessments, as well as reactivity profiling against cysteine surrogates and the validation through differential scanning fluorimetry (DSF) screening against c-Jun N-terminal kinase 3 (JNK3), ubiquitin-specific protease 7 (USP7), and the tumor suppressor p53 core domain followed by intact protein mass spectrometry, was published previously by our group.^46^ This investigation revealed first interesting hits, including the highly cysteine-reactive S_N_Ar-type electrophiles SN001, SN006, and SN007. These hits were then examined further for their potential to rescue the cancer mutant p53-Y220C.^50^,^51^

The tumor suppressor p53 is a central regulator of cell cycle arrest, apoptosis, DNA-repair, and cellular senescence^52–55^ Its inactivation, driven by murine double minute 2 homologue (MDM2) and murine double minute 4 homologue (MDM4) upregulation or missense mutations in the core domain, is a frequent driver of tumorigenesis and chemoresistance^56–61^ These mutations are generally categorized as either DNA-contact alterations (eg R273H) or structural mutations that induce thermal destabilization (eg Y220C).^62–64^ The Y220C mutant, accounting for approximately 100,000 cancer cases annually, is a prime target for pharmacological rescue.^62^,^63^,^65–69^ The mutation creates a solvent-accessible, hydrophobic crevice that lowers the protein’s melting temperature (T_m_) by ~8 °C, causing rapid denaturation.^62–64^,^70^ Because this crevice is distant from the DNA-binding interface, it serves as an ideal pocket for stabilizing ligands. This has led to the development of various binders,^71^,^72^ including the carbazole PK083 (ΔT_m_ = 0.8 °C at 125 μM PK083),^65^ the iodophenole PK5176 (ΔT_m_ = 2.6 °C at 250 μM PK5176),^71^,^73^ (the aminobenzothiazole MB710 (ΔT_m_ = 2.0 °C at 250 μM MB710),^74^ and the potent clinical candidate PC14586 (Rezatapopt).^75^ Notably, the presence of Cys220 in this pocket has also enabled the design of TCIs such as KG13, an acrylamide derivative that restores wild-type stability.^76^ Based on the screening of the first CovLib subset, our group identified multiple S_N_Ar reactive covalent p53-Y220C cleft binders, among them, the highly reactive tyrosine mimicking pyridine derivative SN001 and the pyrazine derivative SN006/7-8 stabilizing the protein up to 4.5 °C and 5.0 °C, respectively.^46^,^50^,^51^

These promising results, considering the small number of compounds in the initial test set, encouraged us to expand the CovLib to a Second Generation Covlib (CovLib Gen2). Hence, we assembled a library of 81 fragments featuring epoxide (EO), vinyl sulfone (VS), acrylamide (AA), and α-cyanoacrylamide (CA) warheads, as well as electron-deficient heteroarenes capable of SɴAr reactions (SN). Herein, we profile the physicochemical properties of the CovLib Gen2 relevant to drug discovery. We initially evaluated solubility through a combination of computational predictions (LogP/LogS) and experimental turbidimetry. Subsequently, we assessed electrophilic reactivity against cysteine surrogates using a high-throughput 5,5’-dithiobis-(2-nitrobenzoic acid) (DTNB) assay, followed by a glutathione (GSH) stability assay to mimic physiologically relevant conditions. We subsequently screened CovLib Gen2 against p53-Y220C via DSF. Hits were validated by time- and concentration-dependent measurements, alongside specificity checks using p53 and a cysteine-light variant of p53-Y220C (p53-Y220C-CL).

## Materials and Methods

### Material

The compounds studied were purchased from Aldrich Market Select (Sigma-Aldrich Chemie GmbH, Taufkirchen, Germany) at purity levels of 90% or higher. Purity was confirmed by high performance liquid chromatography (HPLC) on an Ultimate 3000 HPLC-System with UV-detection (Thermo Fisher Scientific, Dreieich, Germany). Compounds EO004, EO006, EO009, and VS006 were not suitable for HPLC-UV analysis, probably due to a lack of UV absorption. As an alternative, ^1^H nuclear magnetic resonance (NMR) spectroscopy (Bruker Avance III HD 400 MHz) was performed. For compounds EO007, SN034, and SN066, the purity determined did not meet the criteria of ≥ 90% and were therefore excluded from the following investigations. The detected impurities could be caused by degradation during transport or storage at the vendor. Results of the purity confirmation are summarized in Table S 1.1. The corresponding chromatograms, NMR spectra, and chemical shifts are presented in the Figures S 1.1 – S 1.81.

### Structural Filtering Process

The Aldrich Market Select Data Bank (~18 million compounds as of April 2023) was filtered employing a customized python script using the RDKit package. Compounds larger than 22 non-hydrogen atoms and smaller than six non-hydrogen atoms were removed. Further, compounds that show a logP > 2 were excluded. LogP values were calculated by RDKit according to Wildman et al.^77^

### Calculation of Molecular Properties

The SMILES codes of all investigated compounds, including CovLib Generation 1, are listed in Table S 2.1. The molecular properties Heavy Atoms, Molecular Weight, Hydrogen-Bond Donors, Hydrogen-Bond Acceptors, Rotatable Bonds, QPLogS, QPLlogPo/w, and Polar Surface Area of the fragments were calculated using the QikProp module of Schrödinger suite version 2021−1.^78^ Molecules were protonated and preprocessed using Schrödingers’ LigPrep module with default parameters.^78^ All calculations were then carried out using the default parameters and the normal processing mode of QikProp. The results are depicted in Table S 2.2.

### Turbidimetric Solubility Assay

The turbidimetric solubility assay was performed as previously described^46^ using phosphate buffered saline (PBS) pH 7.4 at 25 °C in a 200 μL scale. The 100 mM fragment stocks in dimethyl sulfoxide (DMSO) were diluted by a factor of 4/5 with DMSO in a dilution series. Diluted compounds were added to the buffer, resulting in fragment concentrations of 5 mM, 4 mM, 3.2 mM, 2.56 mM, 2.048 mM, 1.638 mM, 1.311 mM, 1.049 mM, 0.839 mM, 0.671 mM, 0.537 mM, and 0.429 mM and 5% DMSO. Due to lower solubility in DMSO, the compounds AA004, AA005, and SN070 were measured at 2.048 mM as the highest concentration. Extinction at 600–800 nm was measured in a Lumox^®^ multiwell, 96-well-plate (SARSTEDT, Nürnbrecht, Germany) using the CLARIOstar Plate Reader (BMG Labtech, Ortenberg, Germany) for 2 h with 300 rpm double orbital shaking for 60s every 2 min. The concentration-dependent extinction spectra for all compounds, measured at the beginning (0 min), an intermediate cycle (57 min), and at the end (117 min), are depicted in the Figures S 3.1 – S 3.78. The highest concentration up to which no scattering is observed is reported as “minimal instant solubility” (MIS) based on the first measurement cycle and as “minimal final solubility” (MFS) based on the last measurement cycle. MIS and MIC values can be found in Tables 1 and 2.

**Table 1:** Results of the Experimental Turbidimetric Solubility Characterization, Computational QLogPo/w and QPLogS Calculations, and the Reactivity Assessment via GSH and DTNB Assay

Compound	Solubility				Reactivity				
	MIS [mM]	MFS [mM]	QPlogP o/w	QPLogS Predicted Solubility [mM]	t_1/2_ GSH [h]	± SD [h]	t_1/2_ PBS [h]	k_2_ DTNB [M^−1^s^−1^]	± SD [M^−1^s^−1^]
Afatinib					1.1	0.0	>100	n.a. ^a^	
Iodoacetamide					n.a.^b^		n.a.^b^	2.6	0.1
AA001	5.0	5.0	0.65	63	>100		>100	0.018	0.005
AA002	5.0	5.0	2.2	5.0	>100		>100	−0.0033	0.0001
AA003	5.0	5.0	1.1	20	13	0	>100	0.00082	0.00012
AA004	1.0	1.0	1.6	6.5	≪0.033^c^		>100	0.0021	0.0012
AA005	2.0	2.0	1.4	16	n.a.^d^		n.a.^d^	n.a.^d^	
AA006	5.0	5.0	1.6	5.6	>100		>100	0.0078	0.0008
AA007	5.0	5.0	1.5	6.4	2.2	0.0	>100	0.0051	0.0009
AA008	5.0	5.0	0.89	3.0	>100^e^		>100^e^	0.0039^e^	0.0015
CA006	5.0	5.0	0.32	7.1	>100		61	−0.0050	0.0009
CA007	5.0	5.0	0.65	0.23	0.39^f^	0.33	>100	0.0018	0.0012
CA008	2.0	1.6	−0.26	3.6	n.a.^d^		n.a.^d^	n.a.^d^	
EO004	5.0	5.0	0.59	38	n.a.^b^		n.a.^b^	−0.0083	0.0013
EO005	5.0	5.0	1.6	40	20	0	47	0.012	0.001
EO006	5.0	5.0	0.89	107	n.a.^b^		n.a.^b^	−0.024	0.002
EO007	n.a.^c^	n.a.^c^	1.4	111	n.a.^g^		n.a.^g^	n.a.^g^	
EO008	3.2	4.0	−0.27	490	5.2	0.1	5.9	0.0027	0.0008
EO009	5.0	5.0	1.6	15	n.a.^b^		n.a.^b^	−0.022	0.002
EO010	3.2	5.0	1.5	13	30		69	0.013	0.001
SN013	5.0	5.0	1.0	50	>100		>100	−0.0033	0.0012
SN014	3.2	3.2	1.7	16	47	1	88	−0.0047	0.0018
SN015	5.0	5.0	0.70	7.2	>100		>100	0.025	0.001
SN016	1.6	1.6	1.8	11	23	0	>100	0.23	0.01
SN017	2.6	2.6	0.71	12	0.13	0.00	23	0.43	0.00
SN018	5.0	5.0	−0.28	108	n.a.^d^		n.a.^d^	n.a.^d^	
SN019	5.0	5.0	1.2	67	0.81	0.04	>100	1.4	0.0
SN020	5.0	5.0	0.44	59	>100		>100	0.018	0.001
SN021	5.0	5.0	0.44	61	>100^i^		>100	−0.018	0.002
SN022	5.0	5.0	0.91	1.8	≪0.33^h^		>100	0.090	0.002
SN023	5.0	5.0	1.3	12	>100		>100	0.00028	0.00111
SN024	5.0	5.0	−0.39	333	>100		>100	0.018	0.001
SN025	5.0	5.0	−0.30	106	>100		>100	−0.0016	0.0006
SN026	5.0	5.0	0.13	6.5	4.7	0.2	>100	0.039	0.001
SN027	5.0	5.0	0.91	21	>100		>100	0.0019	0.0010
SN028	5.0	2.6	0.91	21	n.a.^d^		n.a.^d^	n.a.^d^	
SN029	1.3	1.3	1.3	14	>100		>100	0.0050	0.0008
SN030	5.0	5.0	1.2	14	2.1	0.2	>100	0.022	0.002
SN031	2.0	2.0	1.2	38	n.a.^d^		n.a.^d^	n.a.^d^	
SN032	2.6	1.3	0.81	1.2	n.a.^d^		n.a.^d^	n.a.^d^	
SN033	2.6	2.6	0.40	5.2	>100		>100	0.026	0.001
SN034	n.a.^c^	n.a.^c^	2.4	0.85	n.a.^g^		n.a.^g^	n.a.^g^	
SN035	5.0	5.0	2.4	0.83	35	0	>100	0.012	0.001

**Notes**: MIS (“minimal instant solubility”) and MFS (“minimal final solubility”) are the highest concentrations up to which no scattering is reported based on the first and the last measurement cycle, respectively. Values for the benchmarks iodoacetamide and Afatinib from previous works.^46^ a no evaluable data obtained. ^b^ The compound was not quantifiable by HPLC-UV. ^c^ At the 0 min time point, no compound was detectable, therefore half-life was estimated to be ≪ 0.033 h. ^d^ 3.33 mM ACN stock not soluble. ^e^ 3.33 mM ACN stock not soluble, the stock was added to the aqueous buffer as a homogenous suspension, and was dissolved in the buffer/organic solvent mixture. ^f ^The compound degrades to ~50% concentration therefore, an adjusted fit function was used. ^g^ The purity of the compound could not be confirmed. It appeared to have been degraded before the studies. ^h^ At the 20 min time point, no compound was detectable, therefore half-life was estimated to be ≪ 0.33 h. ^i^ Relative AUC increases significantly, possibly due to a peak of a degradation product that is not properly separated.

**Table 2:** Results of the Experimental Turbidimetric Solubility Characterization, Computational QLogPo/w and QPLogS Calculations, and the Reactivity Assessment via GSH and DTNB Assay

Compound	Solubility				Reactivity				
	MIS [mM]	MFS [mM]	QPlogP o/w	QPLogS Predicted Solubility [mM]	t_1/2_ GSH [h]	± SD [h]	t_1/2_ PBS [h]	k_2_ DTNB [M^−1^s^−1^]	± SD [M^−1^s^−1^]
SN036	1.0	0.43	2.2	0.58	9.1	0.1	2.9	0.015	0.001
SN037	5.0	5.0	1.0	45	4.2	0.0	>100	0.035	0.001
SN038	5.0	5.0	1.4	11	≪0.033^c^		0.56	0.043	0.000
SN039	5.0	5.0	1.9	0.62	1.6	0.1	>100	0.20	0.00
SN040	2.0	5.0	1.1	4.1	47	1	7.6	−0.0042	0.0011
SN041	4.0	5.0	1.1	4.1	96	4	91	−0.0023	0.0009
SN042	5.0	5.0	0.35	55	3.0	0.2	15	0.028	0.001
SN043	5.0	5.0	1.4	6.8	>100		>100	0.022	0.001
SN044	4.0	3.2	1.8	3.6	n.a.^d^		n.a.^d^	n.a.^d^	
SN045	5.0	5.0	1.4	17	51	10	>100	0.043	0.000
SN046	5.0	5.0	0.67	47	1.5	0.1	>100	0.033	0.001
SN047	5.0	5.0	0.46	643	>100		>100	0.0078	0.0003
SN048	3.2	5.0	0.78	2.0	0.22	0.00	26	0.22	0.00
SN049	5.0	5.0	0.39	6.9	n.a.^d^		n.a.^d^	n.a.^d^	
SN050	5.0	5.0	1.2	3.2	0.14	0.00	44	2.4	0.1
SN051	5.0	5.0	2.4	11	>100		>100	0.014	0.001
SN052	4.0	4.0	1.9	3.5	26	0	>100	0.016	0.000
SN053	4.0	4.0	1.6	14	n.a.^d^		n.a.^d^	n.a.^d^	
SN054	0.67	0.67	0.62	66	0.17	0.00	>100	0.12	0.00
SN055	5.0	5.0	0.45	49	2.1	0.1	>100	0.015	0.00
SN056	5.0	5.0	0.45	50	26	1	4.9	0.015	0.00
SN057	2.6	2.0	1.3	37	>100		>100	0.025	0.000
SN058	5.0	5.0	0.96	111	52	1	>100	0.0022	0.001
SN059	5.0	5.0	0.54	6.8	>100		>100	0.032	0.001
SN060	5.0	5.0	1.5	8.0	0.22	0.00	>100	1.4	0.0
SN061	5.0	5.0	0.56	46	n.a.^d^		n.a.^d^	n.a.^d^	
SN062	4.0	5.0	2.0	1.2	>100		>100	0.027	0.001
SN063	5.0	5.0	0.67	10	8.1	0.2	>100	0.035	0.001
SN064	5.0	5.0	1.3	17	0.59	0.01	>100	0.14	0.00
SN065	5.0	5.0	1.4	11	>100		>100	0.0040	0.0006
SN066	n.a.^c^	n.a.^c^	1.5	35	n.a.^g^		n.a.^g^	n.a.^g^	
SN067	0.67	0.67	1.0	12	1.1	0.0	>100	0.031	0.001
SN068	5.0	5.0	2.7	1.5	24	4	6.4	−0.011	0.001
SN069	5.0	5.0	−0.013	19	≪0.33^h^		5.2	7.2	0.1
SN070	1.6	1.3	0.64	3.8	90	8	>100	0.032	0.001
SN071	5.0	5.0	0.13	103	>100		>100	0.019	0.001
SN072	5.0	5.0	1.39	13	>100		>100	0.0022	0.0008
VS005	3.2	5.0	0.74	29	>100		>100	−0.0080	0.0009
VS006	5.0	5.0	1.7	3.2	n.a.^b^		n.a.^b^	0.0098	0.0012
VS007	5.0	5.0	1.2	9.8	0.070	0.0	>100	0.25	0.00

**Notes**: MIS (“minimal instant solubility”) and MFS (“minimal final solubility”) are the highest concentrations up to which no scattering is reported based on the first and the last measurement cycle, respectively. Values for the benchmarks iodoacetamide and Afatinib from previous works.^46^ b The compound was not quantifiable by HPLC-UV. ^c^ At the 0 min time point, no compound was detectable, therefore half-life was estimated to be ≪ 0.033 h. ^d^ 3.33 mM ACN stock not soluble. ^g^ The purity of the compound could not be confirmed. It appeared to have been degraded before the studies. ^h^ At the 20 min time point, no compound was detectable, therefore half-life was estimated to be ≪ 0.33 h.

### DTNB Assay

The thiol-reactivity assay was performed as previously described^46^ according to the protocol published by Resnick et al^27^ with some modifications.^79^ The cysteine surrogate 2-nitro-5-thiobenzoate anion (TNB^2-^) was prepared in situ by reduction of 5,5′- dithiobis(2-nitrobenzoic acid) (DTNB) in the presence of tris(2-carboxyethyl) phosphine hydrochloride (TCEP). Reaction conditions were 200 μL buffer (20 mM NaPi pH 7.4, 150 mM NaCl), 10% acetonitrile, 100 μM fragment, 25 μM DTNB, and 100 μM TCEP (yielding to 50 μM TNB^2-^) at 37° C. The reaction was performed in a Lumox^®^ multiwell, 96-well-plate (SARSTEDT, Nürnbrecht, Germany) covered with a lid. TNB^2-^ absorbance at 412 nm was monitored every 5 min for 4 h using the CLARIOstar Plate Reader (BMG Labtech, Ortenberg, Germany). Measurements were performed in triplicate, and a parallel experiment without DTNB was conducted to determine the background absorption of the compounds. A measurement without fragment was performed to calculate the extinction coefficient of TNB^2-^ from the absorption of the first time point. The compound background absorbance was subtracted from each measurement, and the remaining TNB^2-^ and compound concentrations were calculated for each time point. The data were then fitted in OriginPro2020 (OriginLab, Northampton, MA, USA) and the second order rate constant k_2_ was calculated using the integrated rate equation of second-order kinetics with multiple reactants:

$${\left[A \right]_t} = {\left[A \right]_0} \cdot {{\left({{{\left[A \right]}_0} - {{\left[B \right]}_0}} \right) \cdot {e^{\left({{{\left[A \right]}_0} - {{\left[B \right]}_0}} \right){k_2}t}}} \over {{{\left[A \right]}_0} \cdot {e^{\left({{{\left[A \right]}_0} - {{\left[B \right]}_0}} \right){k_2}t}} - {{\left[B \right]}_0}}}$$

[A]_0_ and [B]_0_ are the initial concentration of the fragment and TNB^2-^ respectively, and [A]_t_ is the remaining compound concentration as a function of time. The [A]_0_ value for the initial compound concentration was set on the calculated compound concentration at 0 min. [B]_0_ was set by default on the experimentally used concentration of 50 μM. All parameters used for the fitting calculations and the computed results can be found in Table S 4.1 and S 4.2. The k_2_ values are given as the mean value of the triplicate determination with the respective standard deviation, which was calculated according to the rules of error propagation. The corresponding plots are depicted in the Figures S 4.1 – S 4.10.

### Glutathione Assay

The glutathione (GSH) assay was performed as previously described^38^,^46^,^51^ using a method established by Keeley et al^24^ The following reaction conditions were applied: 250 μM fragment, 100 μM ketoprofen or ibuprofen as an internal standard, 5 mM GSH excess, 10% acetonitrile, and phosphate buffered saline (PBS) pH 7.4 at 37 °C. The samples were analyzed on an Ultimate 3000 HPLC-System (Thermo Fisher Scientific, Dreieich, Germany) with UV-detection over a period of 24 h. Highly reactive fragments (half-life t_1/2_ < 1 h) were analyzed every 20 min. The reaction of the compounds with GSH was detected by measuring the decreasing area under the curve (AUC) of the compounds relative to the internal standard. OriginPro2020 (OriginLab, Northampton, MA, USA) was used to fit the relative AUC to the integrated rate equation of pseudo-first order kinetics:

$$relative\ AUC = {e^{ - kt}}$$

For the fitting of the GSH reaction of CA007, an additional +c term was used, because the CA007 concentration approaches ~0.5 instead of zero, possibly due to the reversible reaction.

The half-life t_1/2_ was calculated from the pseudo-first order rate constant k according to the following equation:

$${t_{1/2}} = {{\ln 2} \over k}$$

The GSH measurements were performed in duplicates and multiple runs were averaged using error propagation. In addition, measurements were performed in PBS buffer without GSH to test the hydrolytic degradation. Contrary to the calculations of Keeley et al,^46^ the rate constants k_GSH_ and the corresponding t_1/2_ were not corrected for the degradation reaction in pure buffer For comparison, the half-lives for degradation in PBS (t_1/2_ PBS) are also given. The corresponding degradation plots are presented in the Figures S 5.1 – S 5.10.

### Molecular Biology

For the conducted experiments, the superstable quadruple mutant (M133L/V203A/N239Y/N268D) of the p53 core domain (94–312) T-p53C was used. T-p53C-Y220C additionally contains the investigated Y220C mutation. The cysteine light control variant (T-p53C-Y220C-CL) further has mutations of the surface-exposed cysteines Cys124, Cys182, Cys229, Cys275, and Cys277 to serine residues. The expression and purification of T-p53C (94–312, M133L/V203A/N239Y/N268D) and its mutants using a pET24a(+)_HLT vector was carried out as previously described.^38^ The purity of the expressed protein was monitored by sodium dodecyl sulfate - polyacrylamide gel electrophoresis (SDS-PAGE). The protein sequences of all used constructs are displayed in Table S 6.1.

### Differential Scanning Fluorimetry (DSF)

Differential Scanning Fluorimetry was used to investigate the impact of the compounds on the melting temperatures of T-p53C and its mutants. DSF measurements were performed as previously described^38^,^46^,^51^ using a Qiagen Rotor-Q Model-5-Plex HRM real-time PCR instrument (Qiagen, Hilden, Germany). SYPRO Orange (Life Technologies Corporation, Eugene, OR, USA) served as a fluorescent dye (5x final concentration). A constant heating rate of 270 °C/h was used, while the temperature was raised from 28 °C to 60–70 °C. The excitation and emission filters were set at 470 nm and 610 nm, respectively. The DSF measurements were performed with 8 μM protein in phosphate buffer (25 mM KPi pH 7.2, 150 mM NaCl, 1 mM TCEP, 5% DMSO). For the primary screening, measurements were carried out after 4 and 24 h with final fragment concentrations of 250 and 1000 µM. For concentration-dependent DSF measurements, the compound concentration was varied by diluting the compounds in pure DMSO before added to the protein solution. All samples were incubated at room temperature while shaking. The T_m_ of T-p53C and its mutants was obtained by calculating the maxima of the first derivative of the melting curves using OriginPro2020 (OriginLab, Northampton, MA, USA). Finally, the T_m_ of the protein sample was subtracted from the T_m_ of the sample containing both protein and compound to calculate ΔT_m_. All measurements were performed at least in triplicate and multiple runs were averaged according to the rules of error propagation. The results of the primary screening are depicted in the Tables S 7.1 and S 7.2. Results for the and time and concentration-dependent measurements are presented in the Figures S 7.1 and S 7.2, respectively.

## Results and Discussion

### Selection Process

Our goal was to assemble a library of covalent fragments (CovLib) with a broad range of structural diversity and reactivity. After the initial test set of 20 fragments,^46^ we aimed for expanding the library. The first part of the CovLib consists of four different warhead types to achieve a wide spectrum of covalent binding modes and reactivity. The selected warheads include Michael acceptors, specifically α-cyano acrylamides/acrylates and vinylsulfones. Furthermore, epoxides were added as compact reactive groups featuring two electrophilic centers. Finally, electron-deficient heteroarenes bearing leaving groups capable of undergoing nucleophilic aromatic substitution (S_N_Ar) were selected. The latter type is less studied in drug design but has the advantages of structural rigidity, broad tunability, and, above all, good commercial availability due to its well-established reactions and synthesis in medicinal chemistry.^46^ For this expansion, we decided to add acrylamides as a fifth type of warhead. They are well-studied and already present in covalent-reacting FDA-approved drugs.^7^ Furthermore, they are generally less reactive Michael-type warheads compared to the other two used in this study, and therefore could balance the reactivity spectrum of the library.^80^,^81^

We used the same procedure for selecting the fragments as we did for the original library.^46^ The basis for the selection was the Aldrich Market Select Library (Sigma-Aldrich Chemie GmbH). Compounds larger than 22 non-hydrogen atoms, smaller than six non-hydrogen atoms, and a logP >2 were removed, and structural SMARTs filters were applied.

**Figure 1:**
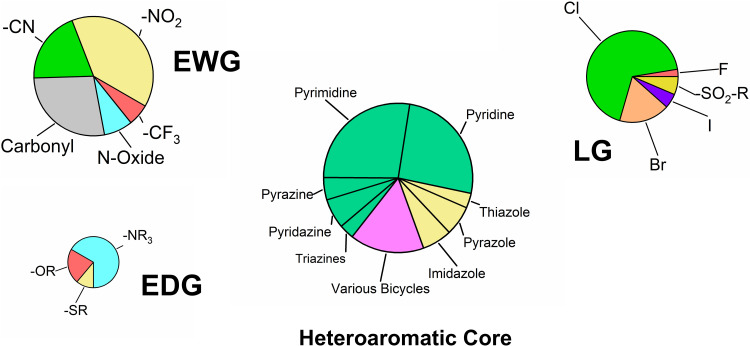
Distribution of the heteroaromatic cores, leaving groups (LG), electron-withdrawing groups (EWG), and electron-donating groups (EDG) of the acquired compounds with S_N_Ar-type warhead. The size of the pie charts reflects the approximate number of substituents. Heteroaromatic cores are color-coded by ring type: six-membered (green), five-membered (yellow), and bicyclic (pink).

The final selection was accomplished by manual choice. In addition to steric and structural features, the assumed reactivity of the compounds was a primary selection criterion. While our initial test set comprised fragments with either very high or low reactivity,^46^ this study specifically targeted compounds exhibiting moderate reactivity. However, the selection process was constrained by commercial availability and cost. Owing to their frequent use as synthetic building blocks, electron-deficient heterocycles are readily available and generally more cost-effective than the other warhead types. Additionally, our group and others have reported the successful application of S_N_Ar-reactive heteroarenes as a viable strategy for covalent FBDD.^23–25^,^30^,^46^,^50^,^51^,^82^,^83^ Consequently, approximately 75% of the acquired fragments are different electron-deficient heteroarenes with diverse leaving groups and various attached electron-withdrawing and donating groups. The distribution of these heteroaromatic scaffolds and respective substitutions is shown in Figure 1.

The selected library consists predominantly of 42 six-membered rings, particularly pyridines and pyrimidines, due to their well-established reactivity profiles.^9^,^84–89^ Additionally, ten five-membered rings such as imidazoles, pyrazoles, and thiazoles were included. These scaffolds typically possess higher electron density and are therefore anticipated to be less reactive than the six-membered analogues.^24^,^90^ Finally, ten bicyclic compounds were acquired to investigate interesting compounds that have been sparsely used as warheads to date, but are very common in approved drugs.^91^

**Figure 2:**
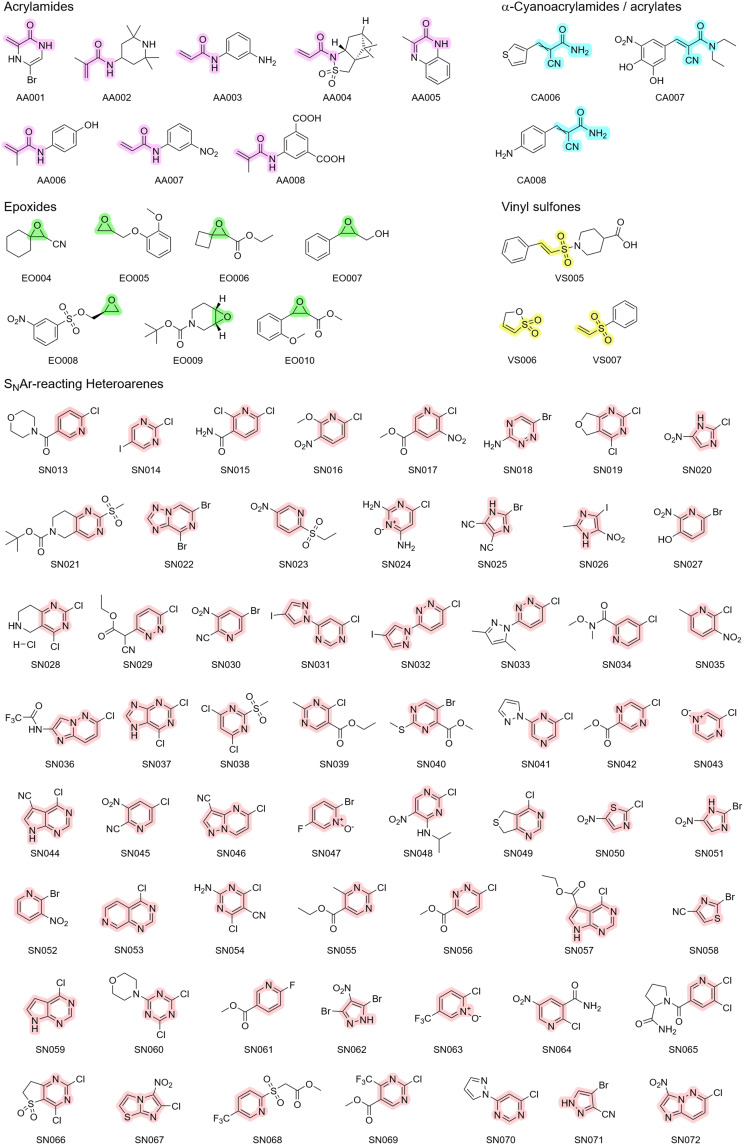
Chemical structures of the fragments purchased for the CovLib Gen2 The scaffolds for the respective warhead types are highlighted in purple for acrylamides, cyan for α-cyanoacrylamides, in green for epoxides, in yellow for vinyl sulfones, and in light red for S_N_Ar-type electrophiles. For clarity, these color assignments are used consistently in all following schemes and illustrations.

Chlorine was selected as the predominant leaving group, offering a theoretical intermediate reactivity within the halide series.^90^ In addition to the four halogens (F, Cl, Br, I), alkyl sulfonyl groups were included to represent highly reactive leaving groups with well-studied reactivity profiles.^86^ It is important to mention that all selected leaving groups also exert an electron-withdrawing effect on the aromatic core. Regarding the substitution pattern, the library turned out to feature a strong preference for electron-withdrawing groups (51 moieties) over electron-donating groups (10 moieties), with a particular emphasis on functional groups with a strong negative mesomeric effect, such as nitro and nitrile moieties. These strong EWGs could enable S_N_Ar reactions at intrinsically more unreactive heteroaromatic scaffolds like five-membered ring systems. In addition to these 60 S_N_Ar-type electrophiles, we purchased eight acrylamides, three α-cyanoacrylamides, seven epoxides, and three vinyl sulfones. The structures of the complete library are depicted in Figure 2.

### Theoretical and Experimental Characterisation of the Library

#### Distribution of Molecular Parameters for Fragment Libraries

**Figure 3:**
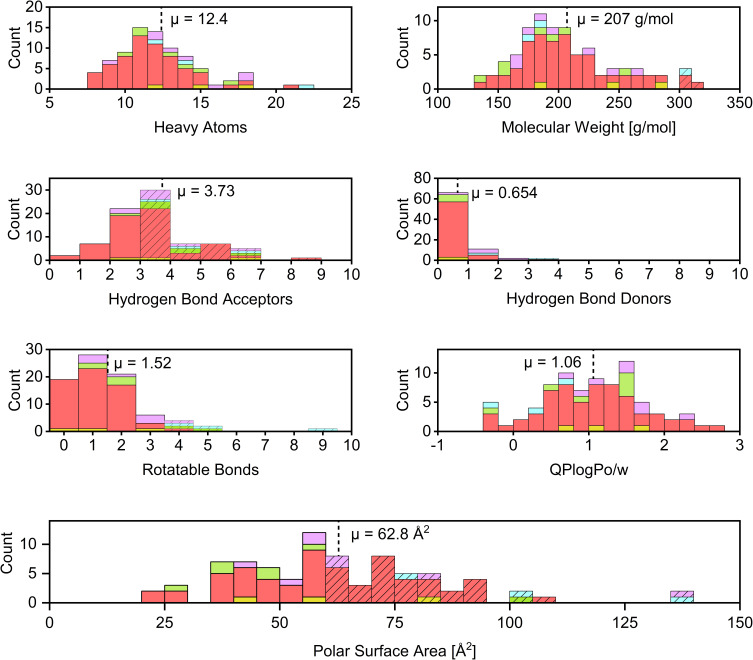
Distribution of heavy atoms, molecular weight, number of hydrogen bond acceptors, number of hydrogen bond donors, number of rotatable bonds, QLogPo/w, and polar surface area. Dashed lines indicate mean values. The hatched bars indicate bins that violate the rule of three. The colours of the bars indicate the respective warhead types. Purple for acrylamides, cyan for α-cyanoacrylamides, green for epoxides, yellow for vinyl sulfones, and light red for S_N_Ar-type electrophiles.

The distribution of molecular parameters, which are often considered crucial for the construction of efficient fragment libraries (rule of three)^47^ is illustrated in Figure 3. Due to the potential for our substructure filters to bias the library toward molecules containing heavy atoms (S, Cl, Br, I), the count of non-hydrogen atoms (heavy atoms) was used as the primary molecular size cutoff criterion instead of molecular weight. Despite this consideration, only four compounds exceeded the 300 g/mol limit, primarily due to the inclusion of heavy halogen atoms. Only three compounds exceeded the threshold of three hydrogen-bond donors, contrasting with ~60% of the fragments violating the threshold for hydrogen bond acceptors. This elevated count could stem from the intrinsic nature of the selected warhead scaffolds, which are rich in heteroatoms that could act as hydrogen bond acceptors (e g, Michael acceptors and electron-withdrawing groups) The majority of compounds also slightly exceed the polar surface area threshold of 60 Å^2^, with a mean of 63.8 Å^2^. High values of these two parameters typically cause higher solubility, which we aimed for. Interestingly, the number of rotatable bonds differs strongly among the warhead classes. The S_N_Ar-type electrophiles have the lowest mean value of 1.10 and thus exhibit high molecular rigidity, which is beneficial for further drug development.^92^

#### Experimental Solubility Assessment

Sufficient solubility is crucial for experimental compound testing and eventual further drug development. Consequently, we used logP values ≤ 2 calculated by RDKit as initial filtering criterion during library assembly. For more sophisticated theoretical characterisation, we calculated the QPLogPo/w and the QPLogS using the Schrödinger QikProp software. Fragment solubility was also experimentally evaluated in PBS buffer (pH 7.4) containing 5% (v/v) DMSO using a turbidimetric assay. Calculated and measured values are presented in Tables 1 and 2, with QPLogPo/w further illustrated in Figure 3. The three compounds SN036, SN054, and SN067 exhibit MIS or MFS values below 1 mM, used for DSF measurements. QPLogS calculations, however, predicted solubility > 1 mM for SN054 and SN067, but not for SN036. Despite the poor measured values, no precipitation was observed during the DSF experiments for any of these compounds. This discrepancy likely arises because the turbidimetric assay is highly sensitive to intrinsic compound absorption, which can interfere with the optical readout and potentially yield falsely low MIS or MIF values despite actual solubility being sufficient.

#### Experimental Reactivity Assessment

**Figure 4:**
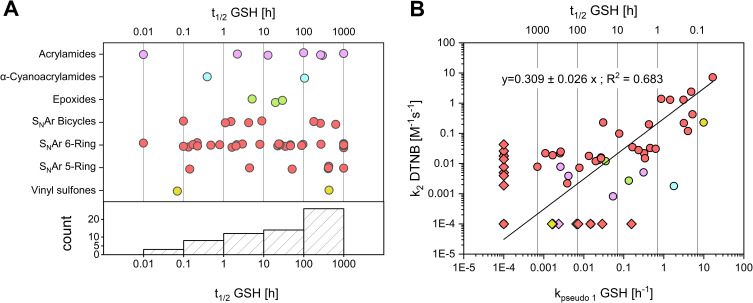
Distribution of experimental thiol reactivites of the CovLib. Data points are color-coded by warhead type: acrylamides (purple), α-cyanoacrylamides (cyan), epoxides (green), vinyl sulfones (yellow), S_N_Ar-type electrophiles (light red). (**A)**: Distribution of half-lives across warhead classes measured via GSH assay. The S_N_Ar warheads are subcategorized into 5-membered, 6-membered, and bicyclic ring systems. Values calculated as >1000 h or <0 h were capped at 1000 h. Compounds undetectable at the first (0 min) or second (20 min) time points were assigned half-lives of 0.01 h or 0.1 h, respectively. (**B**): Correlation between GSH and DTNB reactivity. The line represents a linear regression (R^2^ = 0.683). Kinetic constants below 0.0001 h^−1^ (for GSH assay) or M^−1^s^−1^ (for DTNB assay), or negative values were set to 0.0001 and are depicted as diamonds.

Thiol reactivity of the library was characterized using GSH and TNB^2-^ as cysteine surrogates. The high-throughput DTNB assay was employed as a primary screen, followed by specific validation via a GSH HPLC-UV assay. To control for non-thiol-mediated degradation, reference measurements were performed in nucleophile-free PBS using HPLC. To ensure comparability, iodoacetamide and Afatinib were evaluated as established benchmarks for the DTNB and GSH assays, respectively.^27^,^46^,^79^,^82^,^85^ The determined values are presented in Tables 1 and 2 and illustrated in Figure 4. Corresponding plots and fitting parameters can be found in the Supporting Information.

Linear regression analysis of GSH k_pseudo 1_ versus DTNB k_2_ values revealed a correlation of R^2^ = 0.683 (Figure 4B). A stronger correlation was observed for compounds with high reaction rates compared to more stable fragments. This correlation is regarded as satisfactory considering the known sensitivity limits of plate-based assays for low-reactivity electrophiles.^9^ These data align with previous comparisons of GSH and DTNB assays for acrylamide and chloroacetamide warheads described by Resnick et al^27^ These results underline the potential of the DTNB assay as a primary high-throughput screening method for the estimation of thiol reactivites.

A broad distribution of GSH-reactivities was observed across the entire library and the evaluated warhead classes (Figure 4A), indicating the versatile applicability of CovLib Gen2 for targets with different kinetic requirements. Notably, 12 compounds exhibited mild reactivity (t_1/2_ = 1–10 h); ten of these exhibit S_N_Ar warheads, underscoring their potential as mild arylating agents. Selected findings and insights derived from the extensive GSH reactivity characterization of these diverse warheads are discussed in the following section.

The evaluation of epoxide-based fragments was complicated by handling difficulties related to their partly liquid physical state and the absence of an intrinsic π-system, which hindered accurate quantification via HPLC-UV. While moderate reactivity was observed for the three analyzable compounds (t_1/2_ = 5.2–30 h), notable degradation in PBS was also detected. Consequently, it remained unclear whether compound consumption was driven by nucleophilic substitution at the oxirane ring or by competing hydrolytic pathways, such as (sulfonate) ester hydrolysis. The observed reactivity profiles of the Michael acceptors were generally consistent with previously published trends.^80^,^81^,^93^ The influence of electronic effects was illustrated by the comparison of AA003 and AA007, where the presence of an electron-withdrawing nitro group on the phenyl ring was found to enhance reactivity relative to the amino-substituted analogue. Furthermore, a distinct structure-reactivity relationship was observed for acrylamides and vinyl sulfones, wherein substitution at the α- or β-position induced a substantial reduction in thiol-reactivity, resulting in half-lives exceeding 100 h.

**Figure 5:**
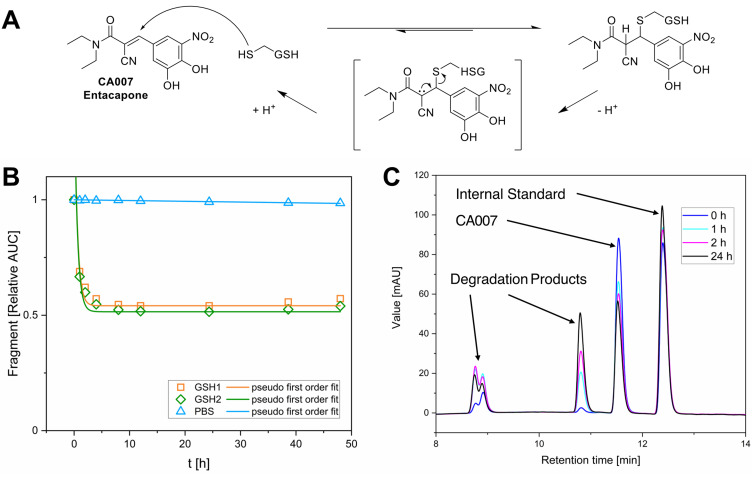
GSH reactivity of CA007. (**A)**: Proposed reaction scheme showing the potential reversible thia-Michael addition of GSH to the α-cyanoacrylamide CA007. (**B)**: Time-dependent depletion of CA007 in the GSH assay monitored by HPLC-UV. Data were fitted using the pseudo-first-order rate equation $relative\ AUC = {e^{ - kt}}$ for PBS and $relative\ AUC = {e^{ - kt}} + c$ for GSH. The constant c was introduced because the CA007 concentration approaches ~0.5 instead of zero, possibly due to the reversible reaction. (**C)**: Excerpt of CA007 GSH assay chromatograms (254 nm) at selected time points.

Despite that, a notable observation was made regarding the α-cyano acrylamide CA007. It should be mentioned that CA007 is the FDA-approved reversible catechol-*O*-methyltransferase (COMT) inhibitor Entacapone, used for the treatment of Parkinson’s disease^94^ and was serendipitously purchased as part of this library. While α-cyanoacrylamides are known for their potential to undergo reversible cysteine addition - driven by β-elimination facilitated through enhanced acidity of the Cα-proton - the experimental verification of this behaviour remains challenging.^2^,^7^ The results obtained from the GSH assay indicate a covalent reversible reaction with GSH. A proposed reaction scheme is shown in Figure 5A. In contrast to other fragments, CA007 was rapidly depleted to ~50% relative AUC and remained at this level over 48 h rather than approaching complete compound consumption (Figure 5B and C). Consequently, we had to adjust the fitting function by adding a constant to the pseudo-first-order rate equation. This adjustment limits the comparability of the calculated t_1/2_-values. We could not find any evidence in the literature regarding a covalent reversible reaction involved in the COMT inhibitory effects of Entacapone.

Compounds SN036, SN040, SN056, and SN068 exhibited shorter half-lives in PBS than in PBS containing 5 mM GSH, indicating intrinsic instability in aqueous buffer rather than S_N_Ar with GSH. Their degradation is likely due to hydrolysis of their esters or trifluoroacetamide moieties. Another explanation is the potential precipitation due to poor solubility in PBS/ACN.

Despite the strong electron-withdrawing nature of N-oxides typically enhancing S_N_Ar reactivity,^95^,^96^ derivatives SN024, SN043, and SN047 were unreactive (t_1/2_ > 100 h). Only SN063 showed mild reactivity (t_1/2_ = 8.1 h), suggesting that N-oxide activation could be a suitable strategy for generating heterocyclic scaffolds with balanced reactivities. Reactivity within five-membered rings varied significantly. While generally less susceptible to S_N_Ar due to higher electron density at the ring carbon atoms, some compounds, like SN026 (t_1/2_ = 4.7 h), were found unexpectedly reactive. Notably, the thiazole SN050 degraded rapidly (t_1/2_ = 0.14h), while the corresponding imidazoles (SN020, SN051) remained inert (t_1/2_ > 100 h), highlighting the higher S_N_Ar reactivity of thiazoles.^83^ Finally, bicyclic compounds displayed relatively unpredictable stability profiles, ranging from complete depletion within 20 minutes (SN022) to high stability of t_1/2_ > 100 h (SN057).

### Biophysical Evaluation of the CovLib Gen2 Against p53 and Its Mutants Using DSF

**Figure 6:**
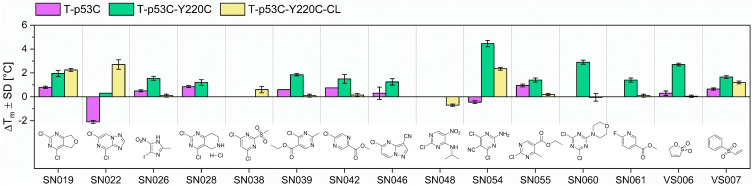
Results of the DSF measurements (1000 µM compound concentration and 24 h incubation time) for the initial fragment hits and their chemical structures. Purple bar: p53 core domain (T-p53C). Green bar: p53 core domain with Y220C mutation. Yellow bar: “cysteine-light” p53 core domain with Y220C mutation and surface-exposed cysteins mutated to serine residues (T-p53C-Y220C-CL).

We evaluated the influence of the CovLib Gen2 compounds on the melting temperature (ΔT_m_) of the p53 mutant T-p53C-Y220C. In the primary screen, we tested all fragments except SN031 and SN032, which were found insoluble under the used conditions. In contrast, the turbidimetric data indicated low but sufficient solubility for SN031 and SN032. Compounds were tested at 250 and 1000 µM with incubation times of 4 and 24 h at RT. A compound was classified as a hit if it induced a thermal shift greater than 1 °C under at least one of the tested conditions. Thermal shifts, for all tested compounds, are reported in the SI. Chemical structures and thermal shifts for the 15 hit compounds are presented in Figure 6. The hits show a strong tendency to S_N_Ar-reacting warheads and vinyl sulfones. This is consistent with previous observations.^46^ The efficacy of these scaffolds is likely due to the nature of the Y220C mutation: the loss of the aromatic tyrosine residue creates a cavity that can be stabilized by the binding of (hetero)aromatic moieties. Furthermore, we observed a preference for compounds with higher thiol reactivity. Notably, all fragment hits exhibited glutathione half-lives of less than 5 h, which is an indicator of covalent fragment binding.

**Figure 7:**
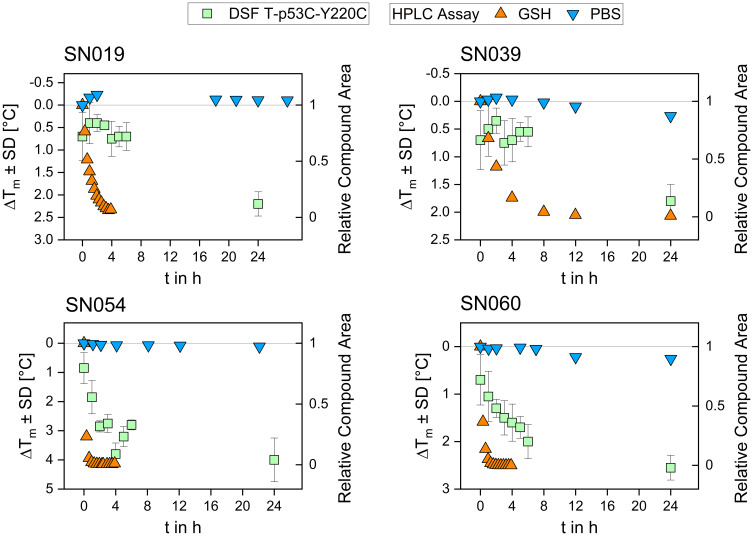
Results for the time-dependent DSF measurement (1000 µM compound concentration) and GSH reactivity assessment for the compounds SN019, SN039, SN054, and SN060. Thermal shifts obtained in the DSF assay are plotted on the left y-axis, while the relative compound area determined in the GSH assay is plotted on the right y-axis.

We investigated the time-dependent stabilization kinetics of all hit compounds using DSF measurements conducted over 24 hours. The time points where thermal stabilization first exceeds 1 °C are presented in Table 3. To compare the kinetic stabilization effect on T-p53C-Y220C with the compounds’ intrinsic thiol reactivity, we plotted the time-dependent GSH depletion alongside the DSF results. Representative plots are shown in Figure 7; the complete data set is provided in the Supporting Information.

**Table 3:** Results of the Time- and Concentration Dependent DSF Measurements. Concentration-Dependent Experiments Were Carried Out After 24 h Incubation Time. Time-Dependent Measurements Were Carried Out with a Fragment Concentration of 1000 µM Except SN028 and SN038, with 250 µM and 500 µM, Respectively, to Obtain Evaluable Melting Curves

Compound	First Measurement Point ΔT_m_ ≥ 1 °C	
	Concentration [µM]	Time
SN019	250	24 h
SN022	62.5	10 min
SN026	None^a^	24 h
SN028	125	24 h
SN038	125	10 min
SN039	500	24 h
SN042	500	24 h
SN046	500	24 h
SN048	None^a^	24 h
SN054	15.63	10 min
SN055	1000	24 h
SN060	31.25	1 h
SN061	None^a^	24 h
VS006	250	24 h
VS007	1000	10 min

**Note**: ^a^ The highest measured stabilization was slightly under 1° C.

Generally, the reaction rates observed with the protein were lower than those with GSH, a difference likely attributable to the experimental temperatures (37 °C for GSH vs. RT for protein incubation). The diagrams reveal a clear correlation: compounds exhibiting rapid GSH consumption (eg SN054 and SN060) showed faster kinetic stabilization of T-p53C-Y220C, whereas fragments with slower GSH reactivity required longer incubation times to achieve maximum thermal stabilization. These results highlight the potential of our standardized GSH assay as a predictive indicator for covalent protein binding kinetics, enabling the early-stage prioritisation of mildly reactive fragments.

**Figure 8:**
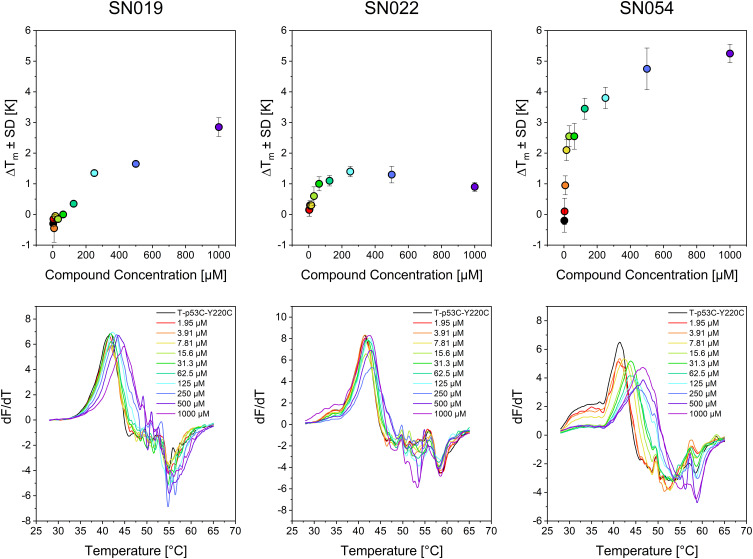
Results of the concentration-dependent DSF measurements after a 24 h incubation time for compounds SN019, SN022, and SN054. The upper diagrams show the thermal shift depending on the respective compound concentration; the lower diagrams show the first derivative (dF/dT) of the melting curves. The colors of the dots and lines indicate the concentrations listed in the lower plots of the melting curves.

To get first insights into reaction selectivity of our hits with T-p53C-Y220C, we performed concentration dependent DSF measurements. The concentrations where thermal stabilization exceeds 1 °C are presented in Table 3. Melting curves and plots of representive fragments are illustrated in Figure 8, the complete data set is provided in the Supporting Information.

Significant stabilization (ΔT_m _≥ 1 °C) at concentrations < 100 µM was observed for only three compounds (SN022, SN054, SN060). At the highest concentrations, however, certain highly reactive compounds (SN022, SN028, SN038, SN060) caused protein destabilization. These effects are linked to multiple arylations at high compound excess, some of which may have destabilizing effects on the protein.^50^

To validate the specificity of fragment binding to Cys220, we performed DSF control experiments using the pseudo-wild type T-p53C and the cysteine-light variant T-p53C-Y220C-CL. In the cysteine-light construct, solvent-accessible cysteine residues are mutated to serine to minimize interactions with competing residues. We hypothesized that a selective Cys220 binder would induce significant thermal shifts in T-p53C-Y220C and T-p53C-Y220C-CL, while showing minimal stabilization of T-p53C, which lacks the target cysteine. DSF results for the control mutants T-p53C and T-p53C-Y220C-CL are presented in Figure 6. The 4,6-dichloro- pyrimidine derivative SN054 induced the highest thermal stabilization of T-p53C-Y220C (ΔT_m_ = 4.45 °C). A substantial shift was retained in the cysteine-light mutant (ΔT_m_ = 2.25 °C), whereas the T-p53C exhibited slight thermal destabilization. These results suggest that while SN054 achieves significant stabilization through specific binding to Cys220, the superior shift observed in T-p53C-Y220C likely results from the additive effect of non-specific arylation of other solvent-exposed cysteines. This hypothesis is supported by the high intrinsic reactivity of the fragment (t_1/2_GSH = 0.17 h). This thermal stabilization of T-p53C-Y220C by SN054 is remarkable regarding the small size of the fragment and comparable to other known strong stabilizing fragments.^50^,^51^ Compound SN019 induced comparable stabilization (ΔT_m_ ~2 °C) in both T-p53C-Y220C and its cysteine-light variant. A minor thermal shift was also observed for T-p53C (ΔT_m_ = 0.8 °C). Structurally, SN019 features a pyrimidine core with two chlorine atoms as well, but with the chlorine leaving groups in 2- and 4-position. SN019 exhibits a milder reactivity profile (t_1/2_ = 0.81 h) compared to SN054.

Compound SN022 displayed a distinct stabilization profile. The cysteine-light mutant (T-p53C-Y220C-CL) exhibited a strong positive shift (ΔT_m_ = 2.7 °C), whereas T-p53C underwent significant destabilization (ΔT_m_ = −2.1 °C). Interestingly, the standard mutant T-p53C-Y220C showed a negligible shift (ΔT_m_ = 0.3 °C). These data suggest a competition between two binding modes: binding to Cys220 stabilizes the protein (as seen in the CL variant), but this effect is likely masked in the T-p53C-Y220C construct by the simultaneous destabilization caused by off-target binding to other solvent-exposed cysteines.

The vinyl sulfone VS007 induced significant thermal stabilization of the cysteine-light mutant (ΔT_m_ = 1.2 °C). However, the compound exhibited excessive glutathione reactivity (t_1/2_ = 0.07 h).

## Conclusion

Following our initial test set, we assembled a second-generation covalent fragment library (CovLib Gen2) of 81 compounds. S_N_Ar-type warheads form the core due to their favorable properties and can alternatively serve as HEFLibs if they lack suitable covalent reactivity. The structural diversity is further expanded by Michael-type electrophiles (acrylamides, α-cyano acrylamides, vinylsulfones) and small epoxides. Although our library is modest in size compared to industrial screening sets, it represents a carefully curated collection tailored to our available academic resources.

To assess intrinsic thiol reactivity, we employed a high-throughput plate-based DTNB assay validated by a subsequent HPLC-UV GSH assay. A linear correlation (R^2^ = 0.683) was found between the two methods, with agreement being stronger for highly reactive compounds. Overall, CovLib Gen2 displayed a broad distribution of reactivities across all warhead classes, confirming its versatility for targets with diverse kinetic requirements. Notably, 12 fragments exhibited desirable mild reactivity profiles (t_1/2_GSH = 1–10 h). We also observed an interesting kinetic profile for the α-cyanoacrylamide CA007, the FDA-approved drug Entacapone. This profile indicates a reversible covalent mechanism in the surrogate assay.

DSF screening against T-p53C-Y220C identified 15 hits, mostly S_N_Ar-reactive heteroarenes and vinyl sulfones. SN054 provided the highest stabilization (ΔT_m_ of up to 4.45 °C). All hits exhibited rapid kinetics (GSH half-lives < 5 h). Time-dependent DSF confirmed that GSH reactivity correlates clearly with protein stabilization kinetics. Selectivity was further probed using T-p53C and the cysteine-light variant T-p53C-Y220C-CL. SN054 retained substantial stabilization (ΔT_m_ = 2.25 °C) in the CL variant but caused slight destabilization in T-p53C.

In summary, screening the second-generation CovLib yielded mildly reactive covalent fragments and identified initial hits against T-p53C-Y220C. These small, structurally diverse scaffolds expand the current landscape of p53-Y220C stabilizers. While their specific effects require further investigation, they serve as excellent starting points for fragment-based optimization.

## Abbreviations

AUC, area under the curve; COMT, catechol-O-methyltransferase; CovLib, covalent fragment library; CRG, covalent reactive group; DMSO, dimethyl sulfoxide; DSF, differential scanning fluorimetry; DTNB, 5,5’-dithiobis-(2-nitrobenzoic acid); FBDD, fragment-based drug discovery; GSH, glutathion; HEFLib, halogen-enriched fragment library; HPLC, high performance liquid chromatography; JNK3, c-Jun N-terminal kinase 3; MDM2, murine double minute 2 homologue;MDM4, murine double minute homologue; MIF, minimal instant solubility; MIF, minimal final solubility; NMR, nuclear magnetic resonance; PBS, phosphate buffered saline; S_N_Ar, nucleophilic aromatic substitution; t_1/2_, half-life; TCEP, tris(2-carboxyethyl) phosphine hydrochloride; TMS, tetramethylsilane; T-p53C, quadruple thermostable mutant of the p53 core-domain, 94–312; TCI, targeted covalent inhibitor; TNB^2-^, 2-nitro-5-thiobenzoate anion; USP7, ubiquitin-specific protease 7.
